# Utilization Probability Map for Migrating Bald Eagles in Northeastern North America: A Tool for Siting Wind Energy Facilities and Other Flight Hazards

**DOI:** 10.1371/journal.pone.0157807

**Published:** 2016-06-23

**Authors:** Elizabeth K. Mojica, Bryan D. Watts, Courtney L. Turrin

**Affiliations:** Center for Conservation Biology, College of William and Mary & Virginia Commonwealth University, Williamsburg, Virginia, United States of America; Pacific Northwest National Laboratory, UNITED STATES

## Abstract

Collisions with anthropogenic structures are a significant and well documented source of mortality for avian species worldwide. The bald eagle (*Haliaeetus leucocephalus)* is known to be vulnerable to collision with wind turbines and federal wind energy guidelines include an eagle risk assessment for new projects. To address the need for risk assessment, in this study, we 1) identified areas of northeastern North America utilized by migrating bald eagles, and 2) compared these with high wind-potential areas to identify potential risk of bald eagle collision with wind turbines. We captured and marked 17 resident and migrant bald eagles in the northern Chesapeake Bay between August 2007 and May 2009. We produced utilization distribution (UD) surfaces for 132 individual migration tracks using a dynamic Brownian bridge movement model and combined these to create a population wide UD surface with a 1 km cell size. We found eagle migration movements were concentrated within two main corridors along the Appalachian Mountains and the Atlantic Coast. Of the 3,123 wind turbines ≥100 m in height in the study area, 38% were located in UD 20, and 31% in UD 40. In the United States portion of the study area, commercially viable wind power classes overlapped with only 2% of the UD category 20 (i.e., the areas of highest use by migrating eagles) and 4% of UD category 40. This is encouraging because it suggests that wind energy development can still occur in the study area at sites that are most viable from a wind power perspective and are unlikely to cause significant mortality of migrating eagles. In siting new turbines, wind energy developers should avoid the high-use migration corridors (UD categories 20 & 40) and focus new wind energy projects on lower-risk areas (UD categories 60–100).

## Introduction

Collisions with anthropogenic structures are a significant and well documented source of mortality for avian species worldwide [1–3]. Collision risk is highest when the flight paths of birds intersect with man-made structures. Collisions of migrating birds with communication towers [2,4], buildings [2], power lines [5–7], and wind turbines [8–12] are well documented in the literature. These studies reported mortalities after the flight hazards were unknowingly installed in bird movement corridors and where limited mitigation measures were available to decrease mortality rates.

Migrating raptors are assumed at the greatest risk of collision when flight hazards, like wind turbines, are concentrated along landscape features attractive to long-distance migrants [13,14]. Raptor migration corridors typically form around leading lines, narrow topographic features like ridgetops and coastlines, which produce updrafts that assist in soaring and gliding flight [15,16]. These same ridgetops and coastlines also produce some of the highest wind power classes utilized for commercial wind power generation [17]. The projected growth of the wind industry and potential increase in impacts on bat and avian species of concern resulted in the formation of federal Land-Based Wind Energy Guidelines to assist wind energy developers and wildlife agencies in assessing and mitigating adverse effects of proposed wind projects [18]. Mitigation includes actions to avoid or minimize impacts, or compensate for impacts to wildlife. A key focus of the guidelines is on site selection because risk to wildlife is not evenly distributed across the landscape and risk can be site- and species-specific [8–10,16].

In the United States, federal guidelines require wind developers to use the best available data on bird species abundance, distribution, and migratory behavior to forecast risk and assist in preliminary site evaluation as part of the Land-based Wind Energy Guidelines and the Eagle Conservation Plan Guidance [18,19]. The guidance documents establish a standardized process for site selection, planning, and pre- and post-construction monitoring. Wind developers and operators are also encouraged to apply for an Eagle Take Permit (50 CFR 22.3) from the U.S. Fish and Wildlife Service to cover liability for potential collisions considered “take” under the Bald and Golden Eagle Protection Act (BGEPA, 16 USC 668-668c).

The bald eagle (*Haliaeetus leucocephalus)* is known to be vulnerable to collision with wind turbines and electrical lines [20–23]. Migrating bald eagles fly 25–600 m above ground height (AGL) [24–26], which is within the rotor swept zone of utility-scale turbines. Although documented turbine collisions in bald eagles through 2016 have been low (*n* = 15) [22], S1 File, collision rates in similar eagle species suggest that the potential for collision is high for bald eagles. Collisions with turbines have been documented in golden eagles (*Aquila chrysaetos*) [11,12,22] and white-tailed eagles (*Haliaeetus albicilla*) [27], species similar to bald eagles in body size, flight style, and foraging techniques. One reason collision risk is so high is the exponential increase in bald eagle populations in many portions of their range, including in the Chesapeake Bay [28]. Currently, few turbines are located in high-use areas for bald eagles, but this is rapidly changing as the both the wind industry and eagle populations expand in the Western Atlantic Flyway.

Researchers have prioritized the development of tools to assess avian risk associated with wind turbines and inform the pre-construction siting process [29]. Species risk and sensitivity maps have been developed for some species [30] but are currently unavailable for migrating bald eagles. To address the need for risk assessment, in this study, we 1) identified areas of northeastern North America utilized by migrating bald eagles, and 2) compared them with high wind-potential areas to identify potential risk of bald eagle collision with wind turbines. Identifying areas with highest potential collision risk could facilitate site selection by wind energy developers and allow the wind energy sector to continue its expansion while minimizing risk to migrating eagles.

## Material and Methods

### Study Area

Our study area included northeastern North America at latitudes between 38°5N and 57°N, including eastern Canada and the New England and Mid-Atlantic bald eagle management units in the United States [31]. The Mid-Atlantic eagle population, estimated at approximately 10,000 individuals (B. Watts unpubl.), is mostly resident with a small portion of juveniles and subadults migrating north to New England and Canada for the summer months [28]. The New England and southern Maritime province population (including New Brunswick, Nova Scotia, and Gaspe Peninsula of Quebec) are roughly estimated at 6,200 individuals based on breeding surveys and survival rates [32–36]. These populations mostly winter in the Mid-Atlantic region, including the Chesapeake Bay [28]. In addition, a third population of unknown size migrates south from northeastern Quebec and western Labrador into New England and the Mid-Atlantic each winter. Eagles migrate along the Western Atlantic Flyway through topographic leading lines (coastlines or mountain ranges) on the Atlantic Coast, Saint Lawrence River, and Appalachian Mountains to reach summering and wintering areas [37].

Potential for wind power generation within the study area is highest in narrow bands of ridgetops in the Appalachian Mountains, Monts Notre-Dame, and Laurentian Mountains and in broader coastal areas along the Atlantic Ocean, Bay of Fundy, and St. Lawrence River/Gulf of St. Lawrence [17,38].

### Telemetry

We captured and marked bald eagles on Aberdeen Proving Ground, Maryland, in the northern Chesapeake Bay between August 2007 and May 2009 [23]. Of the 63 eagles reported in Watts *et al*. [23], 17 migrated north from the Chesapeake Bay within the Western Atlantic Flyway. This included eagles banded as nestlings in Maryland (*n* = 2), eagles banded as nestlings in New York and captured in Maryland during their first winter (*n* = 2), and eagles whose morphometric measurements suggested were from the Chesapeake Bay breeding population (*n* = 3) or northeastern U.S. and Canada breeding populations (*n* = 10) [39–41], Watts unpublished data. Five of the eagles maintained annual summer breeding territories in northern Quebec and Labrador, 52°N-56°N latitude.

Eagles were fitted with 70-g solar-powered global positioning system-platform transmitter terminal (GPS-PTT) satellite transmitters (Microwave Telemetry, Inc. Columbia, MD). Transmitters were programmed to collect GPS location data (±18 m manufacturer estimated error) every hour during daylight and once at midnight. Flight altitude data were not collected. Argos satellites (CLS America, Largo, MD) processed GPS locations and data were archived online by the Satellite Tracking and Analysis Tool [42]. Movement data were preprocessed and formatted by Movebank (www.movebank.org) and downloaded for the analysis. Eagle capture and handling complied with Institutional Animal Care and Use Committee protocols at the College of William and Mary (IACUC20051121-3), Maryland scientific permit 42687, and USGS Bird Banding Laboratory permit 21567.

### Movement Modeling

We identified migration movements for individuals as continuous directional movements north or south ≥100 km (*n* = 132 tracks) [37] and extracted these tracks using ArcMap 10.1 [43]. Two eagles made repeated migratory flights within the same year and season and each of these movements was included as a separate track in the analysis. We used the Move package in R 3.1.2 [44,45] to produce utilization distribution (UD] surfaces for individual migration tracks using the dynamic Brownian bridge movement model (dBBMM) [46]. We set dBBMM parameters to a window size of 17, margin of 7, location error of 18 m, and raster cell size of 1 km. Window size of 17 was based on the maximum number of GPS locations received per day for an individual eagle. The margin was set in proportion to half the window size. Location error was determined by the transmitter manufacturer as ±18 m. We set the cell size to 1 km to generate the most detailed output for the geographic scale of our study area. We excluded one migration track because the number of GPS locations was less than the dBBMM window size of 17. We included approximately 24 hours of additional locations prior to the start and after the end of each migration track to ensure that the entire migration movement was included in the model output. UDs were exported as rasters and overlaid in ArcMap on a grid of 1 km cells (*n* = 1,976,935) that spanned the study area.

We combined UD raster maps produced for individual migration tracks by averaging probabilities for each 1 km cell to create a population-wide UD for the study area [23]. Because the number of locations varied among individual migration tracks, we weighted, combined, and standardized UD surfaces according to the number of locations per track. We chose to weight the UD surfaces based on number of fixes rather than weighting each track equally to relate exposure risk to the amount of time an eagle potentially interacted with a flight hazard. We avoided pseudoreplication by combining all individuals and their tracks to create a single map without comparing tracks to each other. We ordinated UD values per cell and categorized them from highest (top 20% of cells) to lowest use (100% of cells) for display purposes.

### Flight Hazards

We examined the overlap of bald eagle migration movements with wind power density maps to identify locations where estimates of on-shore wind power density (w/m^2^) were available at 50 m AGL [17]. Canadian wind power density maps were not available publically at a scale fine enough to be comparable so we limited the flight hazard analysis to the United States portion of the study area. Wind power classes of 3 or greater were included since these are typically used for planning utility-scale wind facilities. Existing wind turbine locations were plotted on the UD map of eagle migration using available digitized turbine data [47,48]. Locations of existing turbines were validated with high-resolution aerial imagery [Microsoft Bing Maps 2014]. We mapped new turbines not included in U.S. government databases using aerial imagery and ESRI user data in ArcMap 10.1. We identified locations where wind turbines overlapped with areas of high use by migrating eagles to determine sites of highest potential collision risk for eagles.

## Results

### Movement Modeling

**Fig 1:**
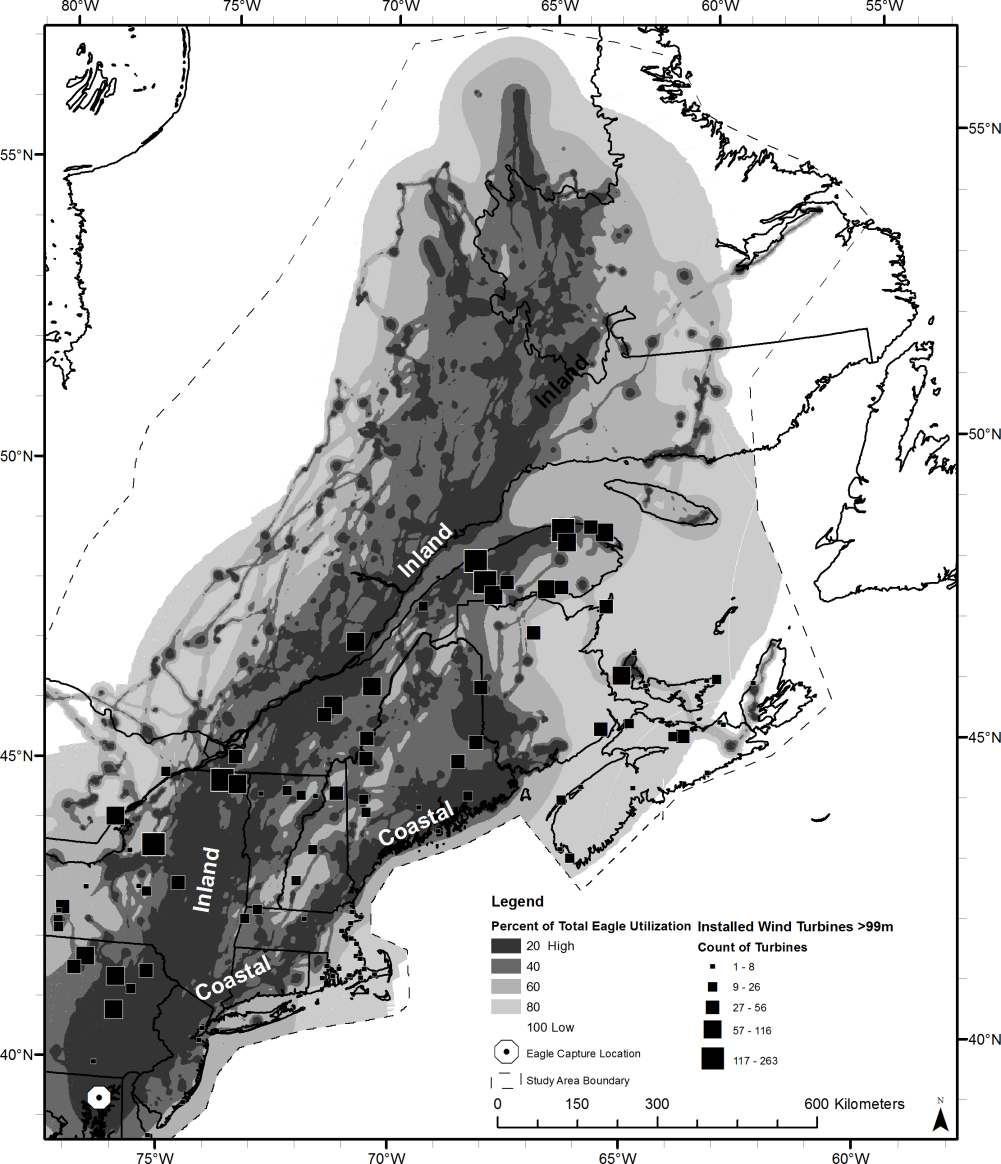
Current wind turbine locations overlaid on utilization distribution map of 17 migrating bald eagles. Bald eagle tracks include 132 migrations from 2007–2014. Darker colors reflect areas with higher eagle utilization.

Seventeen bald eagles migrated during the study period (2007–2014), producing 132 migration tracks in the study area. The number of tracks per eagle ranged from 1–13 and the number of locations per track ranged from 34–1,380 ($\bar{x}$ = 252 ± 19.3 SE). Migration movements were concentrated within two main corridors, one along the Appalachian Mountains (inland corridor, 2,175 km long) and the other along the Atlantic coast (coastal corridor, 1,620 km long; Fig 1). From the northern end of the Chesapeake Bay, a primary movement corridor widens from approximately 70 km to 230 km, stretching from the Atlantic Coast of New Jersey to Harrisburg in the Ridge and Valley region of eastern Pennsylvania. The single corridor then diverges into the inland and coastal corridors in Dutchess County, New York along the Hudson River Valley.

A coastal corridor approximately 50 km wide branches northeast through central Connecticut and Massachusetts until it reaches the Atlantic Coast in New Hampshire. The movement corridor then widens to 90 km along the coast of Maine and into the coast of southern New Brunswick. It turns north-northwest along the Maine-New Brunswick border and ends at the Gaspe Peninsula in Quebec.

The inland corridor is along the Appalachian Mountains in eastern Pennsylvania, New York, Vermont, and into southeastern Quebec. This inland corridor is approximately 145 km wide in northeastern Pennsylvania through northern New York until the corridor splits at Lake Champlain. A short branch of the inland route ends at the St. Lawrence River upstream (west) of Montreal. The inland corridor continues north from Lake Champlain narrows to approximately 70–110 km wide as it continues north into Quebec. At the St Lawrence River, the corridor parallels the northwest coast of the Gulf of St. Lawrence and continues north into Saguenay, Cote-Nord, and ends in Nord du Quebec and western Labrador.

### Flight Hazards

We documented a total of 3,123 wind turbines ≥100 m AGL in the study area, with 1,405 turbines located in the United States and 1,718 in Canada (Fig 1). There were 1,185 turbines (38%) located in UD 20, and 971 turbines (31%) in UD 40. In the United States portion of the study area, commercially viable wind power classes overlapped with 2% of the UD category 20 (i.e., the areas of highest use by migrating eagles) and 4% of UD category 40 (Table 1). The coastal migration route had only 1 concentration of turbines in the UD category 20 (near Bull Hill, Hancock County, Maine) compared to 21 clusters of turbines within the inland route (S1 Table).

**Table 1:** Overlap of wind power class (WPC) and bald eagle utilization distribution (UD) surfaces within the United States portion of the study area. UD categories represent the top 20%, 40%, 60% 80% and 100% of eagle utilization determined from eagle migration data.

Eagle UD Category	Eagle UD Hectares	WPC > = 3 overlap with Eagle UD	No. Installed Turbines
20 (highest)	18,779,858	374,334	568
40	12,657,002	501,646	517
60	5,536,982	259,996	300
80	491,701	120,335	20
100 (lowest)	767	379	0

## Discussion

Predicting potential eagle collision fatalities is a key part of the Eagle Conservation Plan Guidance stage 1 planning process for wind facilities in the United States [19], yet published information on movements of eagle populations is limited. Here we provide a UD map of bald eagle migration corridors in northeastern North America for inclusion in eagle collision risk assessment. This UD map of eagle migration provides the first analysis to evaluate collision risk of migrating eagles over a broad geographic scale and is the first to incorporate eagles of mixed age class, breeding status, and breeding population. The scale and scope of this study can support future assessments of potential impacts of wind energy on migrating bald eagle populations in northeastern North America.

This eagle UD map can be used in planning placement of structures that pose a collision risk to eagles. Site-specific characteristics heavily influence collision risk and predicting flight behavior of eagle migrants could identify potential site conflicts [5]. Bald eagles have been documented colliding with distribution and transmission lines [20,21] and are expected to be most at risk when a flight hazard is not shielded by vegetation, when eagles are distracted during flying (foraging, chasing, or fighting), or during migratory flight [6,20]. We know of no other studies of bald eagles and wind turbine collision risk; however, a recent study documented bald eagles successfully avoiding a new stationary flight hazard erected in a known migratory corridor. In this instance eagles adapted their flight altitudes pre- and post-construction with 96% of eagles flying over a 60 m high transmission line bisecting Kittatinny Ridge, New Jersey [26]. A similar pre- and post-construction study of wind turbines in British Columbia, Canada documented golden eagles detecting and avoiding turbines during migration with fewer flight paths in the collision risk zone after turbines were installed [49]. Bald eagles may also exhibit similar avoidance behavior around turbines, but it has yet to be documented in the literature.

Eagle migration routes described in this study were similar to routes published on juvenile eagles from Labrador [50] and Georgia (S1 File), and juvenile and subadult eagles from Florida [37]. And thus, though our sample size is relatively small we believe our results have broad implications to eagles in the Western Atlantic Flyway. The Chesapeake Bay is a convergence area for bald eagle populations along the flyway supporting 3 distinct populations (northeast, southeast and Chesapeake Bay) throughout the year [28]. Because the Chesapeake Bay acts as an activity hub for migrants on the flyway, we suggest the two northeast migration routes likely represent the main pathways for eagles entering and exiting the Bay region. In addition, eagles migrating through the southern Appalachians use one or more of these routes once they reach Pennsylvania or New York [37], S1 File. We produced a map with greater detail than previous doppler satellite transmitter studies using higher accuracy of the GPS data to define eagle migration corridors. In addition, the broad range of age and breeding populations in our sample of tracked eagles created a comprehensive migration map detailed at the 1 km scale useful for project planning.

Our analysis identified distinct bald eagle migration corridors with limited overlap with commercially viable wind power class areas in northeastern North America. This is encouraging because it suggests that wind energy development can still occur in the study area at sites that are most viable from a wind power perspective and are unlikely to cause significant mortality of migrating eagles. In siting new turbines, wind energy developers may wish to avoid the high-use migration corridors (UD categories 20 & 40) and focus new wind energy projects on lower-risk areas (UD categories 60–100). Our extent of inference is in UD 20 and UD 40 (Fig 1) where we are reasonable certain eagles flew based on the ±18 m accuracy of GPS transmitter locations.

Presumed collision risk was not equal between migratory routes in our study. The coastal route had fewer wind farms than the inland route, which is not surprising since the northeast coast (Delaware to Maine) has fewer areas of commercially viable onshore wind than in the mountains. In coastal areas eagles presumably migrate using thermals, which typically increase flight altitudes over 1,000 m, well out of the rotor-swept zone of turbines and above other flight hazards like communication towers and transmission lines. Eagles using the inland route are primarily using orographic lift, which limits flight to lower altitudes on slopes and ridges where updrafts can subsidize powered flight [51] especially during the cooler fall period when thermals are unavailable [52]. While our transmitter data did not record altitude, we assume bald eagles have similar flight altitude to golden eagles in the study area [51], which have almost identical body size, and flight style, and inland migration route [53]. Based on the number of wind farms currently within the inland migration route and the potential for future construction within available wind power classes ≥3, we believe bald eagles migrating through the inland route are at the highest risk of collision.

Bald eagle collisions have been documented at wind facilities, indicating towers or turbine blades are a new flight hazard for the species. It is unknown whether documented collision rates for bald eagles represent a true low collision risk for the species or are a result of poor carcass retrieval rates in heavily vegetated areas, low carcass searching effort, or both [22]. This level of uncertainty in current collision rates in bald eagles restricts our ability to assess overall collision risk. Refinement of collision rates could be accomplished with sampling designs targeting the 1,185 turbines within the 20 UD. Future studies should increase turbine sample sizes, larger search plots around turbines to search for injured eagles, and longer search intervals during migration periods to better estimate collision and fatality risk to bald eagles during this period. In North America, wind energy is one of the fastest growing energy sources, adding more electricity generating capacity than any other power source in 2013 [54]. Canada currently has 4% (9.6 GW) of its domestic energy from wind and the United States has 2% (65.8 GW) with national capacity goals of 20% by the years 2025 and 2030, respectively [55–59]. The projected growth of this industry includes continued construction of wind farms in northeastern North America [56,58]. We believe the results from this study will be valuable for both planning of future turbine siting and for evaluating collision risk at existing wind facilities. The UD map produced from this study will be made available to planners on the American Wind and Wildlife Institute’s interactive Landscape Assessment Tool http://www.wind.tnc.org/ for preparing risk assessments in the Eagle Conservation Plan Guidance stage 1 planning process [19].

## Supporting Information

S1 File Documentation of personal communications on eagle tracking and eagle fatality data. (PDF)

S2 File Redistribution rights for state and boundary GIS data provided in ArcGIS online. (PDF)

S1 Table Locations of overlap between bald eagle migration corridors and wind turbines in northeastern North America. (XLSX)
