# Supplementary material for: Utilization Probability Map for Migrating Bald Eagles in Northeastern North America: A Tool for Siting Wind Energy Facilities and Other Flight Hazards
Source: PLoS One. 2016 Jun 23;11(6):e0157807. doi: 10.1371/journal.pone.0157807 (PMC4919076; doi:10.1371/journal.pone.0157807)
Supplement: S1 File — (PDF) [file pone.0157807.s001.pdf]

Haram unpubl. data

Dr. Haram is tracking 5 bald eagles fledged from nests in Georgia. Two of the eagles in 2015 migrated up through the southern Appalachian Mountains and used the inland route to the St. Lawrence River and Gaspe Peninsula. Her data can be found online at Movebank.org (Study: Bald Eagle *Haliaeetus leucocephalus* Georgia). Dr. Haram is finishing her PhD work on eagles in 2016 with intent to publish the data in the coming year. EKM is a collaborator on this study and has access to the tracking data to overlay on the UD maps for comparison. Queries on this eagle tracking data can be addressed to: Dr. Brigette Nelson Haram, Graduate Research Assistant, Warnell School of Forestry and Natural Resources, 180 East Green Street, University of Georgia, Athens, Georgia 30602 USA.

US Fish and Wildlife Service, Bald Eagle fatality information through personal communications.

1) US Fish and Wildlife Service, Law Enforcement in Alaska reported 6 fatalities. These are under law enforcement investigation and details of the fatality locations are not releasable as of May 2016. They asked to keep the individual staff names out of the paper and just attribute to the agency. Queries on these fatalities can be addressed to Special Agent in Charge, 1011 East Tudor Road, MS 151, Anchorage, Alaska 99503, USA.
2) Dr. Jeep Pagel, US Fish and Wildlife Service shared knowledge of 3 additional fatalities in the US and Canada not documented in his paper Pagel *et al*. 2013.
    a. 1 Bald Eagle – Erie Shores, Port Burwell, Ontario. Confirmed collision death from turbine ~2009. Source: Ontario Ministry of Natural Resources and Forestry; Bird Studies Canada-Études d'Oiseaux Canada.
    b. 1 Bald Eagle– Castle River Wind Farm, Alberta. Suspected collision death from turbines in 2004. Source: Terrestrial & Aquatic Environmental Managers Ltd. Report "Bird and Bat Interactions With Wind Turbines Castle River Wind Farm, Alberta, 2001-2002".
    c. 1 Bald Eagle – Norfolk. Suspected collision.
