# Supplementary material for: Utilization Probability Map for Migrating Bald Eagles in Northeastern North America: A Tool for Siting Wind Energy Facilities and Other Flight Hazards
Source: PLoS One. 2016 Jun 23;11(6):e0157807. doi: 10.1371/journal.pone.0157807 (PMC4919076; doi:10.1371/journal.pone.0157807)
Supplement: S2 File — (PDF) [file pone.0157807.s002.pdf]

Redistribution Rights

**Description**	**Data Source**	**File Name**	**Directory**	**Redistribution** (see FAQ below)

**World**				
Countries	DeLorme	country.*	\world	Yes 1,2,3
Administrative Units	DeLorme	admin.*	\world	Yes 1,2,3
Airports	DeLorme	airports.*	\world	Yes 1,2,3
Contours	DeLorme	contours.*	\world	Yes 1,2,3
Roads	DeLorme	roads.*	\world	Yes 1,2,3
Railroads	DeLorme	railroads.*	\world	Yes 1,2,3
Continents	ArcWorld Supplement	continent.*	\world	Yes 1,2,3
Regions	ArcWorld Supplement	region.*	\world	Yes 1,2,3
Census IPC Demographics (table)	US Census International Division, CIA Factbook	cntry_ipc.*	\world	Yes 1,2,3
Country Memberships of Political Organizations (table)	CIA Factbook	pol_org.*	\world	Yes 4
Cities	ArcWorld	cities.*	\world	Yes 1,2,3
Populated Places	DeLorme	pop_places.*	\world	Yes 1,2,3
Urban Areas	DeLorme	urban_areas.*	\world	Yes 1,2,3
Gazetteer	DCW	gaz.*	\world	Yes 1,2,3
Linear Water	DeLorme	hydrolines.*	\world	Yes 1,2,3
Water Bodies	DeLorme	hydropolys.*	\world	Yes 1,2,3
Drainage Systems, Lakes, and Rivers	ArcWorld	lakes.*, rivers.*, drainage.*	\world	Yes 1,2,3
World Wildlife Fund Terrestrial Ecoregions	World Wildlife Fund, DCW	wwf_terr.*	\world	No

World Wildlife Fund Marine Ecoregions	World Wildlife Fund, DCW	wwf_mar.*	\world	No
UTM Zones	ArcWorld Supplement, NGA	utmzone.*	\world	Yes 1,2,3
Time Zones	Esri	timezone.*	\world	Yes 1,2,3
Latitude and Longitude Grids	Esri	latlong.*	\world	Yes 1,2,3
Named Latitudes and Longitudes	Esri	geogrid.*	\world	Yes 1,2,3
World Map Background	Esri	world30.*	\world	Yes 1,2,3
**StreetMap North America**				
States and Provinces	TomTom	states.*	\streetmap_na	Yes 1,2
State and Province Boundaries	TomTom	stborder.*	\streetmap_na	Yes 1,2
US Counties	TomTom	counties.*	\streetmap_na	Yes 1,2
US County Boundaries	TomTom	countybnd.*	\streetmap_na	Yes 1,2
City Points	TomTom	cities.*	\streetmap_na	Yes 1,2
Major Cities	TomTom	citiesmjr.*	\streetmap_na	Yes 1,2
City Areas	TomTom	citylim.*	\streetmap_na	Yes 1,2
Postal Points	TomTom	zipcentr.*	\streetmap_na	Yes 1,2
Postal Areas	TomTom	zip5.*	\streetmap_na	Yes 1,2
Maneuvers	TomTom	maneuver.*	\streetmap_na	Yes 1
Highway Exits	TomTom	exits.*	\streetmap_na	Yes 1
Connector Roads	TomTom	connectors.*	\streetmap_na	Yes 1,2
Major Roads	TomTom	mroads.*	\streetmap_na	Yes 1,2
Highways	TomTom	highways.*	\streetmap_na	Yes 1,2
Interstate Highways	TomTom	interstates.*	\streetmap_na	Yes 1,2
Railroads	TomTom	railroads.*	\streetmap_na	Yes 1,2
Lakes	TomTom	lakes_na.*	\streetmap_na	Yes 1,2
Rivers	TomTom	rivers.*	\streetmap_na	Yes 1,2
Water Polygons	TomTom	waterp.*	\streetmap_na	Yes 1,2

Major Water Polygons	TomTom	waterpmjr.*	\streetmap_na	Yes 1,2
Airports	TomTom	airports.*	\streetmap_na	Yes 1,2
Institutions	TomTom	institutions.*	\streetmap_na	Yes 1,2
Large Area Landmarks	TomTom	landmarks.*	\streetmap_na	Yes 1,2
Parks	TomTom	parks.*	\streetmap_na	Yes 1,2
Recreation Areas	TomTom	recarea.*	\streetmap_na	Yes 1,2
Transportation Terminals	TomTom	transterm.*	\streetmap_na	Yes 1,2
Retail Centers	TomTom	retail_center.*	\streetmap_na	Yes 1,2
Cartographic Streets	TomTom	streetscarto.*	\streetmap_na	Yes 1
Detailed Streets	TomTom	streets.*	\streetmap_na	Yes 1

**United States**				
States and Counties (generalized)	ArcUSA, US Census, Esri	states.*, counties.*	\usa\census	Yes 1,2,3
States and Counties	Esri, derived from TomTom, US Census	dtl_st.*, dtl_cnty.*	\usa\census	TomTom - Yes 1,2; US Census - Yes 1,2,3; Esri - Yes 1,2,3
State and County Boundaries	Esri, derived from TomTom	dtl_st_ln.*, dtl_cty_ln.*	\usa\census	TomTom - Yes 1,2
County Population Estimates (tables)	US Census, Federal State Cooperative Program for Population Estimates	popestmt90.*, popestmt00.*	\usa\census	Yes 4
Census Tracts	TomTom, US Census, Esri	tracts.*	\usa\census	TomTom - Yes 1,2; US Census - Yes 1,2,3; Esri - Yes 1,2,3
Census Feature Class Codes (table)	US Census	cfcc.dbf	\usa\census	Yes 4
111th Congressional Districts	Esri	cd111.*	\usa\census	Yes 1,2,3
112th Congressional	Esri	cd112.*	\usa\census	Yes 1,2,3

Districts				
Cities	US Census	cities.*	\usa\census	Yes 4
Populated Place Points	US Census	places.*	\usa\census	Yes 4
Populated Place Areas	TomTom, US Census	placeply.*	\usa\census	Yes 1,2
Core Based Statistical Areas	TomTom	cbsa.*	\usa\census	Yes 1,2
Highways	Esri	highways.*	\usa\trans	Yes 1,2,3
Major Highways	Esri	mjr_hwys.*	\usa\trans	Yes 1,2,3
National Transportation Atlas - Interstate Highways	US Bureau Transportation Statistics	intrstat.*	\usa\trans	Yes 4
National Transportation Atlas - Railroads	US Bureau Transportation Statistics	rail100k.*	\usa\trans	Yes 4
Census Urbanized Areas	US Census	urban.*	\usa\census	Yes 4
Major Parks	National Park Service, ArcUSA, TomTom	parks.*	\usa\landmarks	Yes 1,2
Drainage Systems, Lakes, and Rivers (generalized)	ArcWorld	drainage.*, lakes.*, and rivers.*	\usa\hydro	Yes 1,2,3
Telephone Area Code Boundaries	TomTom	areacode.*	\usa\census	Yes 1,2
ZIP Code Points	TomTom, US Census, Esri	zip_usa.*	\usa\census	TomTom - Yes 1,2; Esri - Yes 1,2,3; US Census - Yes 1,2,3
ZIP Code Areas (Five-Digit)	TomTom, US Census, Esri	zip_poly.*	\usa\census	TomTom - Yes 1,2; Esri - Yes 1,2,3; US Census - Yes 1,2,3
ZIP Code Areas (Three-Digit)	Esri, derived from TomTom	zip3.*	\usa\census	TomTom - Yes 1,2; Esri - Yes 1,2,3; US Census - Yes

				1,2,3
National Atlas - Airports	National Atlas of the United States	airports.*	\usa\trans	Yes 4
National Atlas - Cities	National Atlas of the United States	cities_dtl.*	\usa\census	Yes 4
National Atlas - Urbanized Areas	National Atlas of the United States	urban_dtl.*	\usa\census	Yes 4
National Atlas - Federal and Indian Land Areas	National Atlas of the United States	fedlandp.*	\usa\landmarks	Yes 4
National Atlas - Federal Land Lines	National Atlas of the United States, USGS	fedlandl.*	\usa\landmarks	Yes 4
National Atlas - Water Feature Areas and Lines	National Atlas of the United States, USGS	hydroply.*, hydroln.*	\usa\hydro	Yes 4
National Atlas - Public Land Survey	National Atlas of the United States, USGS	publdsur.*	\usa\other	Yes 4
National Atlas - Historic Earthquakes	National Atlas of the United States, USGS	quakehis.*	\usa\landmarks	Yes 4
National Atlas - Volcanoes	Smithsonian Institution, Global Volcanism Program, National Atlas of the United States	volcano.*	\usa\landmarks	Yes 4
Airports	TomTom	airportp.*	\usa\trans	Yes 1,2
Institutions	TomTom	institut.*	\usa\landmarks	Yes 1,2
Large Area Landmarks	TomTom	lalndmrk.*	\usa\landmarks	Yes 1,2
Parks	TomTom	park_dtl.*	\usa\landmarks	Yes 1,2
Recreation Areas	TomTom	recareas.*	\usa\landmarks	Yes 1,2
Transportation Terminals	TomTom	tranterm.*	\usa\trans	Yes 1,2
Geographic Names Information System - Buildings	USGS - GNIS	gblding.*	\usa\landmarks	Yes 4
Geographic Names Information System - Cemeteries	USGS - GNIS	gcemetry.*	\usa\landmarks	Yes 4

Geographic Names Information System - Churches	USGS - GNIS	gchurch.*	\usa\landmarks	Yes 4
Geographic Names Information System - Golf Locales	USGS - GNIS	ggolf.*	\usa\landmarks	Yes 4
Geographic Names Information System - Hospitals	USGS - GNIS	ghospitl.*	\usa\landmarks	Yes 4
Geographic Names Information System - Locales	USGS - GNIS	glocale.*	\usa\landmarks	Yes 4
Geographic Names Information System - Populated Places	USGS - GNIS	gppl.*	\usa\landmarks	Yes 4
Geographic Names Information System - Schools	USGS - GNIS	gschools.*	\usa\landmarks	Yes 4
Geographic Names Information System - Summits	USGS - GNIS	gsummit.*	\usa\landmarks	Yes 4
State Plane Zones (NAD 1927, NAD 1983)	NOAA, USGS, Esri	spcszn27.*, spcszn83.*	\usa\other	Yes 1,2,3
USGS Topographic Quadrangle Series Indexes - 1:24,000, 1:100,000, 1:250,000	ArcUSA	topoq24.*, topoq100.*, topoq250.*	\usa\other	Yes 1,2,3
Census Block Groups	TomTom, US Census, Esri	blkgrp.*	\usa\census	TomTom - Yes 1,2; US Census - Yes 1,2,3; Esri - Yes 1,2,3
Census Block Centroid Populations	US Census	blockpop.*	\usa\census	Yes 1,2
Rivers and Streams	USGS, Esri	dtl_riv.*	\usa\hydro	Yes 1,2,3
Water Bodies	USGS, Esri	dtl_wat.*	\usa\hydro	Yes 1,2,3
**Europe**				

Europe Demographic - NUTS 0 Demographics	Michael Bauer Research GmbH	nuts0.*	\europe\data	Yes 1
Europe Demographic - NUTS 1 Demographics	Michael Bauer Research GmbH	nuts1.*	\europe\data	Yes 1
Europe Demographic - NUTS 2 Demographics	Michael Bauer Research GmbH	nuts2.*	\europe\data	Yes 1
Europe Demographic - NUTS 3 Demographics	Michael Bauer Research GmbH	nuts3.*	\europe\data	Yes 1

All data are available for internal use.

Frequently Asked Questions (FAQ)

Q: Are all of the sample data provided on the Esri Data & Maps disks freely redistributable?

A: No. Much of the sample data are provided by multiple, third party data vendors under license to Esri for inclusion on these disks specifically for use with Esri software.

Q: What does the last column “Redistribution” on the Redistribution Rights matrix with its “Yes” or “No” answers mean?

A: Each data vendor has its own data licensing policies and may grant varying redistribution rights to end users. Please consult the Redistribution Rights Codes below to determine the redistribution rights for a certain sample data file provided on the CD–ROMs and DVD-ROMs. As used herein, “Geodata” shall mean any digital data set consisting of geographic data coordinates and associated attributes.

“No"   Internal Use Only. No redistribution rights are granted by the data vendor and the data is for the end user's own internal use only.

“Yes 1"   Redistribution rights are granted by the data vendor for hard-copy renditions or static, electronic map images (e.g. .gif, .jpeg, etc.) that are plotted, printed, or publicly displayed with proper metadata and source/copyright attribution to the respective data vendor(s).

“Yes 2"   Geodata are redistributable with a Value-Added Software Application developed by Esri Business Partners on a royalty-free basis with proper metadata and source/copyright attribution to the respective data vendor(s).

“Yes 3"   Geodata are redistributable without a Value-Added Software Application (i.e., adding the sample data to an existing, [non]commercial data set for redistribution) with proper metadata and source/copyright attribution to the respective data vendor(s).

“Yes 4"   Public domain data from US government are freely redistributable with proper metadata and source attribution.

Q: Are there any legal terms and conditions I need to be aware of under this license to use the sample data provided on the Esri Data & Maps disks?

A: Yes. The terms and conditions below apply to all the sample data sets provided on the disks.

High Risk Activities: (a) The Software, Data, and Documentation are not fault-tolerant and are not designed, manufactured, or intended for use or resale for insurance underwriting or with critical health and safety or online control equipment in hazardous environments that require fail-safe performance, such as in the operation of nuclear facilities, aircraft navigation, or communication systems, air traffic control, emergency response, terrorism prevention or response, life support, or weapons systems ("High Risk Activities"). ESRI SPECIFICALLY DISCLAIMS ANY EXPRESS OR IMPLIED WARRANTY OF FITNESS FOR HIGH RISK ACTIVITIES.

(b) To the extent permitted by law, Licensee agrees to indemnify, defend, and hold Esri, its officers, directors, employees, agents, subcontractors, licensors, successors, and assigns harmless from and against any and all liability, losses, claims, expenses (including attorneys' fees), demands, or damages of any kind, including direct, indirect, special, punitive, incidental, or consequential damages, arising out of or in any way connected with Licensee's use or permitting the use by others of the Software, Data, and vendor's hardware for High Risk Activities. Delivery of the Software, Data, and vendor's hardware does not constitute a waiver of the rights and obligations set forth in this Article.

Proprietary Rights and Copyright: Licensee acknowledges that the Data and Related Materials contain proprietary and confidential property of Esri and its licensor(s). The Data and Related Materials are owned by Esri and its licensor(s) and are protected by United States copyright laws and applicable international copyright treaties and/or conventions.

Limited Warranty and Disclaimer: Esri warrants that the media upon which the Data and Related Materials are provided will be free from defects in materials and workmanship under normal use and service for a period of ninety (90) days from the date of receipt.

THE DATA AND RELATED MATERIALS ARE EXCLUDED FROM THE LIMITED WARRANTY, AND THE LICENSEE EXPRESSLY ACKNOWLEDGES THAT THE DATA CONTAIN SOME NONCONFORMITIES, DEFECTS, OR ERRORS. ESRI DOES NOT WARRANT THAT THE DATA WILL MEET LICENSEE'S NEEDS OR EXPECTATIONS; THAT THE USE OF THE DATA WILL BE UNINTERRUPTED; OR THAT ALL NONCONFORMITIES, DEFECTS, OR ERRORS CAN OR WILL BE CORRECTED. ESRI IS NOT INVITING RELIANCE ON THESE DATA, AND THE LICENSEE SHOULD ALWAYS VERIFY ACTUAL DATA.

EXCEPT FOR THE LIMITED WARRANTY SET FORTH ABOVE, THE DATA AND RELATED MATERIALS CONTAINED THEREIN ARE PROVIDED "AS-IS," WITHOUT WARRANTY OF ANY KIND, EITHER EXPRESS OR IMPLIED, INCLUDING, BUT NOT LIMITED TO, THE IMPLIED WARRANTIES OF MERCHANTABILITY AND FITNESS FOR A PARTICULAR PURPOSE.

Exclusive Remedy and Limitation of Liability: The entire liability of Esri or its licensor(s) and Licensee's exclusive remedy shall be to terminate the Agreement upon Licensee returning the Data and Related Materials to Esri with a copy of Licensee's invoice/receipt and Esri returning the license fees paid to Licensee.

IN NO EVENT SHALL ESRI AND/OR ITS LICENSOR(S) BE LIABLE FOR COSTS OF PROCUREMENT OF SUBSTITUTE GOODS OR SERVICES; LOST PROFITS, LOST SALES, OR BUSINESS EXPENDITURES, INVESTMENTS, OR COMMITMENTS IN CONNECTION WITH ANY BUSINESS; LOSS OF ANY GOODWILL; OR FOR ANY INDIRECT, SPECIAL, INCIDENTAL, EXEMPLARY, OR CONSEQUENTIAL DAMAGES ARISING OUT OF THIS AGREEMENT OR USE OF THE DATA AND RELATED MATERIALS, HOWEVER CAUSED, ON ANY THEORY OF LIABILITY, AND WHETHER OR NOT ESRI OR ITS LICENSOR(S) HAVE BEEN ADVISED OF THE POSSIBILITY OF SUCH DAMAGE. THESE LIMITATIONS SHALL APPLY NOTWITHSTANDING ANY FAILURE OF ESSENTIAL PURPOSE OF ANY EXCLUSIVE REMEDY.

Third Party Beneficiary: Esri's licensor(s) has (have) authorized Esri to (sub)distribute and (sub)license its (their) data as incorporated into the Data and Related Materials. As an intended third party beneficiary to this Agreement, the Esri licensor(s) is (are) entitled to directly enforce, in its own name, the rights and obligations undertaken by the Licensee and to seek all legal and equitable remedies as are afforded to Esri.

Q: How should I attribute the sample data provided on the CD–ROMs and DVD-ROMs if I use it as proscribed above?

A: In the event that the data vendor(s) has (have) granted the end user permission to redistribute the Geodata, please use proper proprietary or copyright attribution for the various data vendor(s), and provide the associated metadata file(s) with the Geodata. In

compliance with FGDC metadata standards, Esri has attempted to practice proper metadata methodologies by providing any data source information, descriptions, and file names to assist in this effort.
